# NAD+ Metabolism Regulates Preadipocyte Differentiation by Enhancing α-Ketoglutarate-Mediated Histone H3K9 Demethylation at the PPARγ Promoter

**DOI:** 10.3389/fcell.2020.586179

**Published:** 2020-11-24

**Authors:** Keisuke Okabe, Allah Nawaz, Yasuhiro Nishida, Keisuke Yaku, Isao Usui, Kazuyuki Tobe, Takashi Nakagawa

**Affiliations:** ^1^Department of Molecular and Medical Pharmacology, Faculty of Medicine, University of Toyama, Toyama, Japan; ^2^First Department of Internal Medicine, Faculty of Medicine, University of Toyama, Toyama, Japan; ^3^Department of Endocrinology and Metabolism, Dokkyo Medical University, Tochigi, Japan; ^4^Research Center for Pre-Disease Science, University of Toyama, Toyama, Japan

**Keywords:** NAD+, nampt, alpha-ketoglutarate, demethylation, adipocyte, preadipocyte, differentiation, metabolomics

## Abstract

Obesity has become a serious problem in public health worldwide, causing numerous metabolic diseases. Once the differentiation to mature adipocytes is disrupted, adipocyte hypertrophy and ectopic lipid accumulation leads to the inflammation in adipose tissue and systemic metabolic disorders. Intracellular metabolic state is known to change during cell differentiation and it affects the cell fate or the differentiation through epigenetic mechanism. Although the mechanism of preadipocyte differentiation has been well established, it is unknown how metabolic state changes and how it affects the differentiation in predipocyte differentiation. Nicotinamide adenine dinucleotide (NAD+) plays crucial roles in energy metabolism as a coenzyme in multiple redox reactions in major catabolic pathways and as a substrate of sirtuins or poly(ADP-ribose)polymerases. NAD+ is mainly synthesized from salvage pathway mediated by two enzymes, Nampt and Nmnat. The manipulation to NAD+ metabolism causes metabolic change in each tissue and changes in systemic metabolism. However, the role of NAD+ and Nampt in adipocyte differentiation remains unknown. In this study, we employed liquid chromatography-mass spectrometry (LC-MS)- and gas chromatography-mass spectrometry (GC-MS)-based targeted metabolomics to elucidate the metabolic reprogramming events that occur during 3T3-L1 preadipocyte differentiation. We found that the tricarboxylic acid (TCA) cycle was enhanced, which correlated with upregulated NAD+ synthesis. Additionally, increased alpha-ketoglutarate (αKG) contributed to histone H3K9 demethylation in the promoter region of PPARγ, leading to its transcriptional activation. Thus, we concluded that NAD+-centered metabolic reprogramming is necessary for the differentiation of 3T3-L1 preadipocytes.

## Introduction

Obesity is a global epidemic that causes numerous metabolic diseases, such as type 2 diabetes, dyslipidemia, hypertension, and cardiovascular disease. The pathophysiology of obesity consists of hyperplasia and hypertrophy of adipocytes. Hyperplasia is driven by the differentiation of preadipocytes in the stromal vascular fraction in adipose tissue into mature adipocytes (Rodeheffer et al., 2008; Rutkowski et al., 2015). Previous studies have revealed that adipocyte hyperplasia leads to the healthy expansion of adipose tissue; however, when preadipocyte differentiation is disrupted, adipocyte hypertrophy and ectopic lipid accumulation occur in inflammation and metabolic disorders (Klöting and Blüher, 2014; Vishvanath and Gupta, 2019). Therefore, elucidating the mechanism underlying the preadipocyte differentiation into mature adipocytes is essential to provide a new approach to combat obesity-based metabolic diseases.

Previous studies have established that the cascade of transcription factors centered around PPARγ and C/EBPα plays a crucial role in preadipocyte differentiation (Farmer, 2006). It is also known that metabolic reprogramming accompanies cellular differentiation (Roberts et al., 2009). Although metabolic changes were previously considered a consequence of differentiation, recent studies have revealed that metabolic changes are actually an independent regulator of cellular differentiation (Folmes et al., 2011; Takubo et al., 2013; Xu et al., 2013; Mathieu et al., 2014; Prigione et al., 2014). Mass spectrometry-based metabolomics enable the measurement of even very low levels of intermediate metabolites, including those produced by glycolysis, the pentose phosphate pathway, the tricarboxylic acid (TCA) cycle, and nicotinamide adenindinucleotide (NAD+) metabolism (Chiarugi et al., 2012). Although a number of omics-based studies have been performed to determine the metabolic changes that occur during the differentiation of 3T3-L1 preadipocytes, few have focused on the role of NAD+ metabolism during differentiation (Roberts et al., 2009; Nagai et al., 2011).

NAD+ is a coenzyme implicated in many cellular redox reactions and connects nutrient status to energy production (Houtkooper et al., 2010; Chiarugi et al., 2012). Additionally, NAD+ acts as a substrate for poly(ADP-ribose)polymerases (PARPs) and class III NAD+-dependent deacetylases known as sirtuins, and plays key roles in a diverse array of biological processes (Imai and Guarente, 2014). In mammalian cells, NAD+ is predominantly synthesized through the salvage pathway, in which nicotinamide phosphoribosyltransferase (Nampt) is a rate-limiting enzyme (Houtkooper et al., 2010). Nampt catalyzes the formation of nicotinamide mononucleotide (NMN) from nicotinamide (NAM) and 5′-phosphoribosyl-pyrophosphate (PRPP); NMN is subsequently converted to NAD+ by nicotinamide mononucleotide adenylyltransferase (Nmnat) (Ruggieri et al., 2015). NAD+ synthesis and the subsequent activation of sirtuins are considered to be important events that enable cellular adaptation to meet energy demands (Chen et al., 2008; Song et al., 2014). Previous studies have demonstrated that deletion of murine Nampt in mature adipocytes causes multi-organ insulin resistance and adipose tissue dysfunction (Stromsdorfer et al., 2016). However, another study demonstrated that mature adipocyte-specific Nampt knockout mice exhibited resistance to diet-induced obesity and had improved insulin resistance (Nielsen et al., 2018). Thus, the role of NAD+ synthesis in cellular and systemic metabolism appears to be more complex than previously assumed and may vary between physiological and pathological conditions. More importantly, the role of NAD+ synthesis in adipocyte differentiation is not fully understood. Nmnat1 and PAPR1 have been reported to play crucial roles in the early stage of preadipocyte differentiation, and Nmnat2 has been found to support the enhanced energy demands required to sustain differentiation (Ryu et al., 2018). However, it is unclear how the enhanced NAD+ biosynthesis regulates the later stage of adipocyte differentiation.

In this study, we employed LC-MS (liquid chromatography-mass spectrometry)- and GC-MS (gas chromatography-mass spectrometry)-based targeted metabolomics to elucidate the metabolic changes that occur during 3T3-L1 preadipocyte differentiation. This approach revealed that Nampt-mediated NAD+ biosynthesis plays a critical role in metabolic reprogramming during differentiation. Enhanced TCA cycle activity leads to the epigenetic regulation of 3T3-L1 preadipocyte differentiation through α-ketoglutarate (αKG)-dependent demethylation of H3K9me3 in the *Pparg* promoter region. Furthermore, we demonstrated that dietary supplementation of αKG prevents diet-induced obesity in mice by promoting appropriate adipogenesis. Our study uncovers a new role for NAD+ biosynthesis in adipogenesis and proposes a novel therapeutic approach to combat obesity.

## Materials and Methods

### Cell Culture

3T3-L1 preadipocytes were obtained from JCRB Cell Bank (National Institute of Biomedical Innovation, Japan). Cells were maintained in Dulbecco’s modified Eagle’s medium (DMEM) with 10% fetal bovine serum (FBS) (Gibco). To induce differentiation, 2 days post-confluent 3T3-L1 preadipocytes (designated as day 0) were fed DMEM containing 10% FBS, 1 μg/mL insulin, 1 μM dexamethasone, and 0.5 mM 3-isobutyl-1-methylxanthine (Takara Bio) for 2 days. Then, the medium was replaced with medium containing 1 μg/ml insulin. After day 5, cells were maintained in medium without insulin, which was changed every 2 days.

3T3-L1 cells were treated with 100 nM FK866 with or without 100 μM NMN to inhibit Nampt. αKG supplementation was achieved using 5 mM dimethyl alpha-ketoglutarate (DM-αKG) (Tokyo Chemical Industry). To inhibit the histone lysine demethylases (KDMs), 1 μM JIB-04 (Cayman Chemical) was used. All PARP inhibitors, AG-14361 (ADooQ), UPF-1069 (SANTA CRUZ), and Olaparib (ADooQ) were added at the indicated concentration after the induction of differentiation and were maintained for 5 days.

For Oil Red O staining, cells were fixed with 10% formalin after being washed with PBS. Fixed cells were then washed with distilled water, followed by washing with 60% isopropanol, and then dried. Dried samples were stained with Oil Red-O solution. Stained samples were washed with ddH2O prior to observation. The absorbance of Oil Red O was detected with a U-5100 spectrophotometer (HITACHI) to quantify lipid accumulation.

### Animals

C57BL/6J mice were fed a normal chow diet and maintained under a 12 h light-dark cycle. High fat high sucrose diet (Research Diet) with or without 1.5% DM-αKG (Tokyo Chemical Industry) in drinking water was initiated when mice were 6 weeks old. Body weight and food intake was measured once a week. The intraperitoneal glucose tolerance test (ipGTT) was conducted after 10 weeks of high fat high sucrose diet feeding. The mice were fasted for 16 h beforehand and injected with glucose (1 g/kg body weight) intraperitoneally. The blood was collected from the tail vein and the blood glucose concentration was measured using an automatic blood glucose meter (NOVA Biomedical). The intraperitoneal insulin tolerance test (ipITT) was conducted after 16 weeks of high fat high sucrose diet feeding. After 4 h of fasting, mice were injected with 1.0 units/kg body weight human insulin intraperitoneally. The blood collection and the measurement of the concentration of blood glucose level were performed as described above. All mice experiments were approved by the Animal Experiment Committee of the University of Toyama.

### Western Blotting

Cells were lysed with RIPA buffer (150 mM NaCl, 1.0% NP-40, 1 mM EDTA, 0.1% SDS, 0.1% sodium deoxycholate, and 50 mM Tris–HCl, pH 8.0) and lysates were subjected to western blotting. Antibodies used for western blotting include Nampt (Bethyl, Cat# A300-372A, Dilution 1:1000), PAR (TREVIGEN, Cat# 4336-BPC-100, Dilution 1:1000), β-actin (Wako, 017-24551, Dilution 1:500), Histone H3 (Cell signaling, Cat# 4499, Dilution 1:2000), and Histone H3K9Ac (Cell Signaling, Cat# 9649, Dilution 1:2000). Anti-mouse Nmnat1 rabbit polyclonal antibody (Dilution 1:1000) was raised against the synthetic peptide corresponding to mouse Nmnat1 residues 130–146. HRP-conjugated secondary antibodies were obtained from Millipore. PVDF membranes (Millipore) were used for blotting. Signals were detected and quantified using an LAS4000 mini digital imager (GE Health Care) and ImageQuant TL (GE Health Care), respectively.

### Real-Time Quantitative PCR

Total RNA was extracted from 3T3-L1 cells using TRI Reagent (Molecular Research Center, Inc.). cDNA was synthesized using ReverTra Ace qPCR RT Master Mix with gDNA Remover (Toyobo, Japan) according to the supplier’s protocol. Real-time PCR was performed with THUNDERBIRD SYBR qPCR Mix (Toyobo) on a Thermal Cycler Dice Real Time System II (Takara Bio). Data was quantified using the 2-ΔΔCt method, and Rpl13a was used as an internal control for normalization. qPCR primers are listed in Supplementary Table 1.

### Metabolite Extraction

Metabolites were extracted from cells with water/methanol/chloroform (25:25:50 by volume). After centrifugation, the aqueous phase was isolated and dried using a SpeedVac SPD1010 (Thermo). For LC-MS analysis, the dried sample was reconstituted with 50 μl LC-MS grade water (Wako) and filtered through a 0.45 μm Millex filter unit (Millipore). For GC-MS analysis, two-step derivatization was carried out. First, carbonyl functional groups were protected by methoximation using 20 μL of 20 mg/mL methoxyamine hydrochloride in pyridine at 30°C for 90 min. Next, samples were derivatized using 90 μL of N-methyl-N-trimethylsilyltrifluoroacetamide with 1% trimethylchlorosilane (MSTFA + 1% TMCS, Pierce) at 37°C for 30 min.

### Metabolomic Analysis by LC-MS and GC-MS

Polar metabolite levels were determined by multiple reaction monitoring (MRM) mode using an Agilent 6460 Triple Quad mass spectrometer coupled to an Agilent 1290 HPLC system. Chromatographic conditions were used as previously described (Hikosaka et al., 2014). Organic acids and amino acids were assayed by selected ion monitoring (SIM) mode using an Agilent 5977 MSD Single Quad mass spectrometer coupled to an Agilent 7890 Gas chromatograph. For GC conditions, a 30 m long DB5-MS column with a 10 m Duragard precolumn was used with a 0.25 mm diameter and 0.25 μm film thickness. A constant flow rate of 1.1 mL/min helium was used as the carrier gas. The temperature program started at 60°C for 1 min, increased at 10°C/min to 325°C, and held at 325°C for 10 min. The injector and detector temperatures were set at 290°C. Metabolite levels were semi-quantified by calculating the integrated sum of the area.

### Chromatin Immunoprecipitation

Chromatin immunoprecipitation was performed using a SimpleChIP^®^ Enzymatic Chromatin IP Kit (Magnetic Beads) (CST) according to the manufacturer’s protocol. Briefly, the complexes of chromatin and DNA were obtained from 3T3-L1 cells on day 4 after inducing differentiation. Cells were crosslinked by incubation in 1% formaldehyde for 10 min, after which crosslinking was quenched with glycine. Cells were collected into tubes after being washed with PBS and were then incubated with buffer A + DTT + PIC. DNA was fragmented by adding buffer B + DTT + micrococcal nuclease and incubating for 20 min at 37°C with mixing every 5 min until EDTA was added to stop the digestion. DNA fragment length was confirmed to be approximately 150–900 bp by electrophoresis with a 1% agarose gel. Cell nuclei were centrifuged and resuspended in ChIP buffer + PIC. Nuclei were fractured using a sonicator (VP-050, TAITEC) set to 25% output for 20 s for 10 cycles. Next, this chromatin solution was subjected to chromatin immunoprecipitation. The solution was incubated with ChIP grade Histone H3K9me3 antibody (Abcam, Cat# ab8898) overnight at 4°C on a rounding rotator. Chromatin/antibody complexes were precipitated with protein G magnetic beads. Then, the crosslinking was reversed and the DNA was purified. The primers used in following qPCR were designed for C/EBP binding site on *Pparg* promoter region located in the upstream of TSS site and were listed in Supplementary Table 1 (Meruvu et al., 2011).

### Analysis of Energy Metabolism

The energy metabolism of 3T3-L1 cells treated with FK866 with or without NMN was analyzed using a Seahorse XFe96 Analyzer and FluxPak-XFe96 (Agilent Technologies) according to the supplier’s protocol. Briefly, 3T3-L1 preadipocytes were seeded at 5000 cells per well into FluxPak-XFe96 and were cultured to induce differentiation in 2 days after cells became confluent. 3T3-L1 cells were shifted to Seahorse XF assay media which is free of sodium bicarbonate and were incubated for 1h at 37°C in a non-CO2 incubator before the measurements on day 5 after the induction. Cellular oxygen consumption rate (OCR) measurements were obtained at the baseline and following injection of oligomycin (1 μM), FCCP (1 μM), and antimycin A plus rotenone (AA/ROT, 1 μM). Non-mitochondrial oxygen consumption was defined as minimum rate measurement after rotenone/antimycin A injection. Basal respiration was calculated subtracting non-mitochondrial oxygen consumption from the last rate measurement before the injection of oligomycin. ATP production was calculated subtracting minimum rate measurement after oligomycin injection from the last rate measurement before the injection of oligomycin. Maximal respiration was calculated subtracting non-mitochondrial oxygen consumption from maximum rate measurement after FCCP injection. Spare capacity was calculated subtracting basal respiration from maximal respiration. For measurement of the extracellular acidification rate (ECAR), the assay medium was supplemented with glutamine (1 mM). The measurements were performed at the baseline and after injection of glucose (10 mM), oligomycin (1 μM), and 2-DG (100 mM). At least five independent basal measurements were performed for each assay. Three measurements were performed after each injection, with each measurement consisting of a 10-s mixing period and 3-min measurement period, in accordance with the manufacturer’s instructions.

### Histology

For hematoxylin and eosin (H&E) staining, epididymal white adipose tissue (eWAT) and inguinal white adipose tissue (iWAT) were immediately soaked in 4% paraformaldehyde (PFA) after dissection. Paraffin-embedded tissues were cut into 5-μm thick slices and mounted on slides. Sections were subjected to H&E staining as follows. After the deparaffinization, slides were dipped into H&E counter stain for 2–4 min and washed with running water. Then, they were put in warm water to remove color, followed by kishirin washing steps, and then cover slips were applied. Images were acquired using an Olympus BX61 microscope, 10× objective. Adipocyte size was calculated from randomly selected cells from 200× magnification fields of view using ImageJ v1.52 (NIH) (Schneider et al., 2012).

### Small Interfering RNA

For the small interfering RNA (siRNA) treatment, 3T3-L1 preadipocytes were seeded in 6 well plates at 1.5 × 10^5/well and cultured 24 h with DMEM containing 10% FBS. Then, the cells were transfected with siRNAs using 5 μL Lipofectamine^®^ RNAiMAX (Thermo Fisher Scientific) and 5 pmol siRNA in 500 μL of Opti-MEM (Thermo Fisher Scientific). All the siRNA, siGENOME Mouse Sirt1 (Cat# M-049440-00-0005), siGENOME Mouse Sirt6 (Cat# M-061392-00-0005), siGENOME Mouse Sirt7 (Cat# M-054325-01-0005), and siGENOME Control Pool Non-targeting siRNA (Cat# D-001206-13-20), were obtained from Dharmacon. The treated cells were incubated for 2 days and then, were subjected to the subsequent experiments.

### Statistical Analysis

Statistical significance was assessed using two-tailed student’s *t*-test and one-way ANOVA with the *post hoc* Tukey test, calculating standard deviations of the mean. Significant differences in all figures are indicated with single, double, or triple asterisks when the *p*-value is <0.05, <0.01, or <0.005, respectively.

## Results

### Upregulation of NAD+ Biosynthesis Through the Salvage Pathway Is Required for 3T3-L1 Preadipocyte Differentiation

**FIGURE 1:**
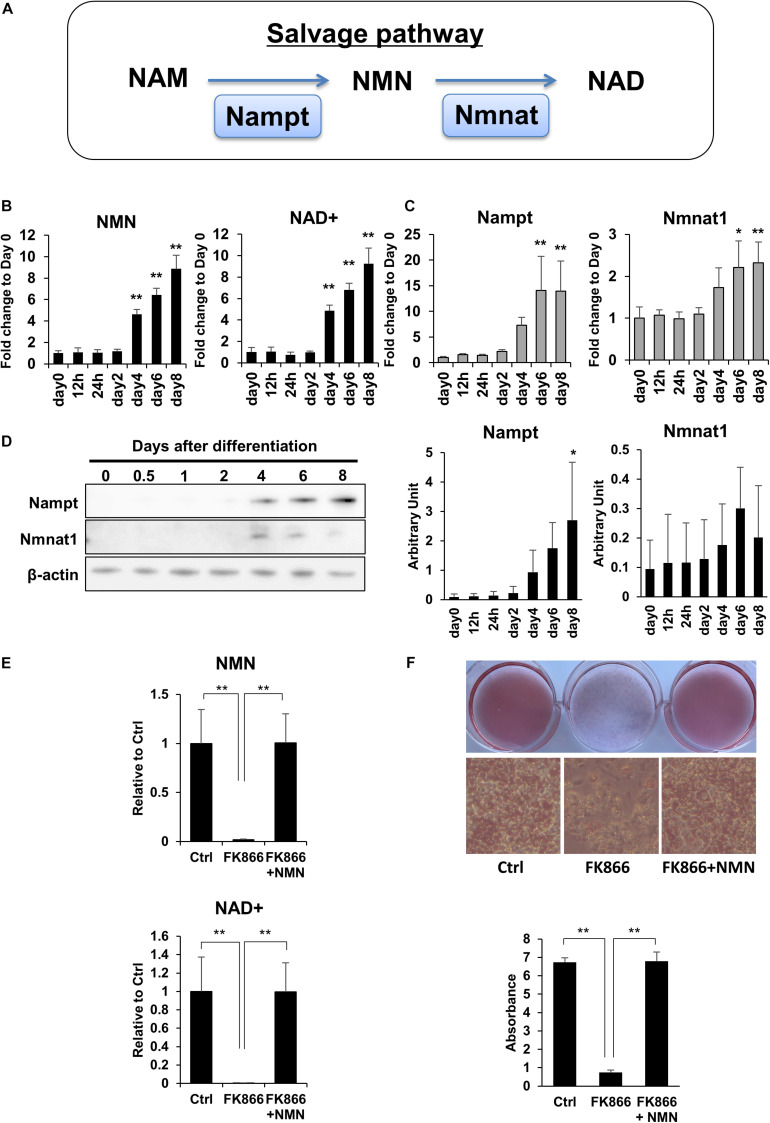
Upregulation of NAD+ biosynthesis in the salvage pathway is required for 3T3-L1 preadipocyte differentiation. **(A)** Schematic of NAD synthesis in the salvage pathway. **(B)** Intracellular levels of NMN and NAD+ were measured with LC-MS during the differentiation of 3T3-L1 cells (*n* = 3). Data are represented as mean ± SD. **(C)** Relative expression levels of enzymes in the NAD+ synthetic pathway during the differentiation of 3T3-L1 cells (*n* = 3). Data are represented as mean ± SD. **(D)** Western blotting for enzymes in the NAD+ synthetic pathway during the differentiation of 3T3-L1 cells. The representative image from three independent experiments. The signals of Nampt and Nmnat1 were quantified and adjusted with those of β-actin (*n* = 3). **(E)** 3T3-L1 cells were treated with 100 nM FK866 or 100 nM FK866 and 100 μM NMN during differentiation. Intracellular levels of NMN and NAD+ were measured on day 4 after inducing differentiation (*n* = 4). Data are represented as mean ± SD. **(F)** Oil Red-O staining of differentiated 3T3-L1 cells treated with 100 nM FK866 or 100 nM FK866 and 100 μM NMN on day 8 after inducing differentiation. The lipid accumulation was quantified as absorbance. (*n* = 4) Ctrl represents control. *p < 0.05, **p < 0.01.

To investigate the role of NAD+ metabolism in preadipocyte differentiation, we induced the differentiation of 3T3-L1 preadipocytes and performed NAD metabolome analysis using LC-MS. In mammalian cells, NAD+ is primarily synthesized through the salvage pathway in which Nampt converts NAM to NMN, which is then converted to NAD+ by Nmnat (Figure 1A). We found that NAD+ and NMN levels were significantly increased during the differentiation of 3T3-L1 cells (Figure 1B). Correlating with the levels of NAD+ and NMN, gene expression levels of Nampt and Nmnat1, a nuclear isozyme of Nmnat, began increasing approximately 4 days after inducing differentiation (Figure 1C). Consistently, the protein expression of Nampt also increased approximately 4 days after inducing differentiation (Figure 1D). These data suggest that NAD+ synthesis in the salvage pathway is upregulated during 3T3-L1 preadipocyte differentiation.

Next, we wondered if the increase in NAD+ level is required for preadipocyte differentiation. To address this, we used FK866, a highly specific chemical inhibitor of Nampt (Hasmann and Schemainda, 2003) to block NAD+ production during the differentiation of 3T3-L1 cells. As shown in Figure 1E, FK866 treatment significantly suppressed the rise of both NMN and NAD+ levels following the induction of differentiation. We also found that the reduced NAD+ level caused by FK866 treatment significantly prevented the differentiation of 3T3-L1 cells, as demonstrated by Oil Red O staining (Figure 1F). Moreover, NMN supplementation during FK866 treatment completely restored both the decreased NAD+ level and the differentiation of 3T3-L1 cells (Figures 1E,F). These data demonstrate that increased NAD+ production through the salvage pathway is required for 3T3-L1 preadipocyte differentiation.

### Upregulation of NAD+ Synthesis Promotes Energy Metabolism During 3T3-L1 Preadipocyte Differentiation

NAD+ plays an important role in energy production as a coenzyme in several redox reactions. In glycolysis, NAD+ is reduced to NADH by glyceraldehyde 3-phosphate dehydrogenase (GAPDH), and NADH is oxidized to NAD+ by lactate dehydrogenase in anaerobic glycolysis. In mitochondria, NAD+ is reduced to NADH by three redox reactions in the TCA cycle. NADH is then oxidized to NAD+ in the mitochondrial electron transport chain to efficiently produce ATP. Therefore, we investigated if upregulation of NAD+ synthesis promotes energy metabolism during adipogenesis.

**FIGURE 2:**
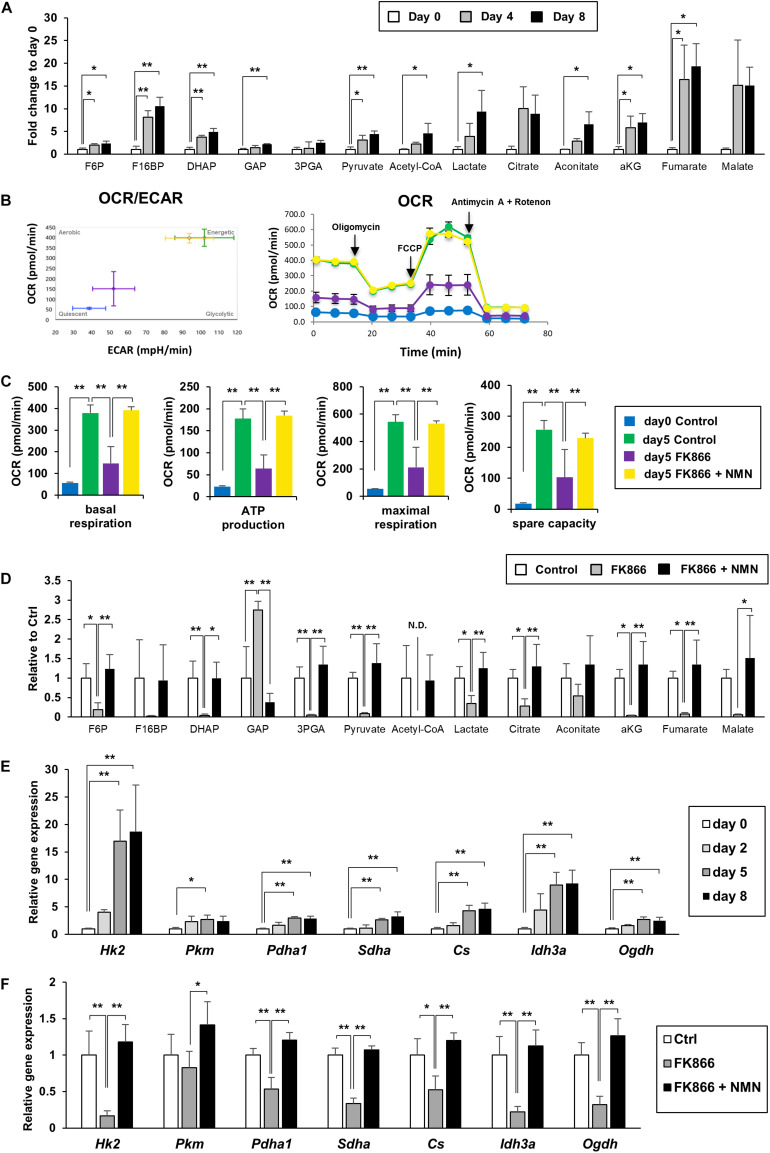
Upregulation of NAD+ synthesis promotes energy metabolism during 3T3-L1 preadipocyte differentiation. **(A)** Metabolites of glycolysis and the TCA cycle in 3T3-L1 cells were measured with LC-MS or GC-MS during the differentiation (*n* = 3). Data are represented as mean ± SD. Each abbreviation represents as follows, F6P: Fructose 6-phosphate, F16BP: Fructose 1,6-bisphosphate, DHAP: Dihydroxyacetone phosphate, GAP: Glyceraldehyde 3-phosphate, 3PGA: 3-phosphoglycerate, aKG: alpha-ketoglutarate. **(B,C)** Oxygen comsumption rate (OCR) and extracellular acidification rate (ECAR) were measured with flux analyzer on day 5 during the differentiation of 3T3-L1 cells treated with 100 nM FK866 or 100 nM FK866 and 100 μM NMN **(B)**. Basal respiration, ATP production, maximal respiration, and spare capacity were calcurated from the difference of OCR, injecting Oligomycin, FCCP, and Antimycin A + Rotenon **(C)**. (*n* = 6) Data are represented as mean ± SD. **(D)** Metabolites of glycolysis and the TCA cycle on day 4 during the differentiation of 3T3-L1 cells treated with 100 nM FK866 (*n* = 4) or 100 nM FK866 and 100 μM NMN. Data are represented as mean ± SD. **(E)** Relative expression levels of enzymes in glycolysis and the TCA cycle during the differentiation of 3T3-L1 cells. (*n* = 4) Data are represented as mean ± SD. **(F)** Relative expression levels of enzymes in glycolysis and the TCA cycle on day 5 of the differentiation of 3T3-L1 cells treated with 100 nM FK866 or 100 nM FK866 and 100 μM NMN (*n* = 4). Data are represented as mean ± SD. *p < 0.05, **p < 0.01.

We measured the metabolites involved in glycolysis, the pentose phosphate pathway, and the TCA cycle using LC-MS and GC-MS. We discovered that the levels of most metabolites in glycolysis, the pentose phosphate pathway, and the TCA cycle were significantly increased during the differentiation of 3T3-L1 cells, demonstrating that energy metabolism was enhanced (Figure 2A). We also employed a flux analyzer to examine how the metabolic state of those pathways was altered during differentiation. Interestingly, both extracellular acidification rate (ECAR) and OCR were increased as 3T3-L1 cells differentiated (Figure 2B). These results suggest that both anaerobic glycolysis and aerobic ATP synthesis in mitochondria were enhanced during differentiation. Additionally, suppression of NAD+ synthesis by FK866 treatment impeded these increases, which could be rescued by NMN supplementation (Figures 2B,C). Consistent with these data, our metabolomic analysis revealed that the level of each metabolite in glycolysis and the TCA cycle was reduced by FK866 treatment and was reconstructed by NMN supplementation (Figure 2D). Furthermore, gene expression of the enzymes involved in glycolysis and the TCA cycle was also increased after inducing differentiation, but was suppressed by FK866 treatment (Figures 2E,F). Additionaly, supplementation of NMN with FK866 could rescue the expression of these genes (Figure 2F). Together, our data indicate that glycolysis and the TCA cycle were promoted during 3T3-L1 preadipocyte differentiation, and enhanced NAD+ synthesis was required for these metabolic changes.

### NAD+ Biosynthesis Through the Salvage Pathway Regulates Adipogenic Gene Expression

**FIGURE 3:**
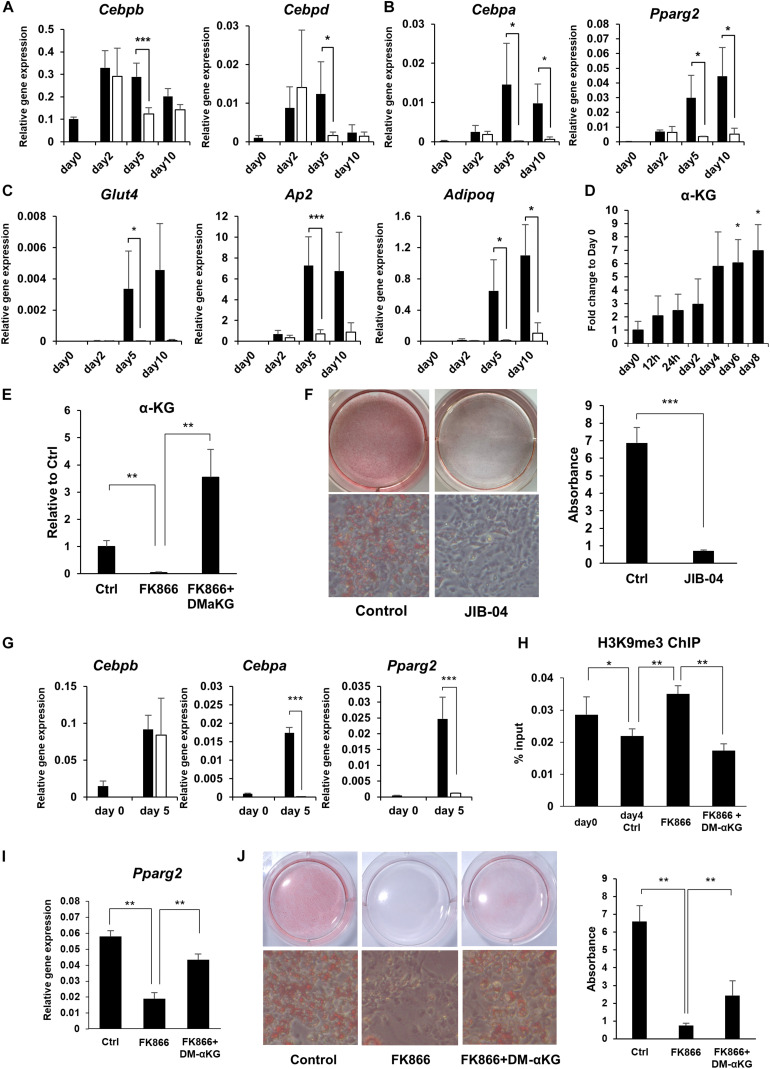
NAD+ biosynthesis in the salvage pathway regulates adipogenic gene expression. **(A–C)** Relative expression levels of genes related to adipogenesis during the differentiation of 3T3-L1 cells treated with 100 nM FK866 (*n* = 3). Data are represented as mean ± SD. The black bars represent control and the white bars represent FK866. **(D)** α-KG levels during the differentiation of 3T3-L1 cells measured with GC-MS (*n* = 3). Data are represented as mean ± SD. **(E)** α-KG levels on day 5 of differentiation of 3T3-L1 cells treated with 100 nM FK866 or 100 nM FK866 and 5 mM dimethyl alpha-ketoglutarate (DM-αKG) (*n* = 4). Data are represented as mean ± SD. **(F)** 3T3-L1 cells treated with JIB-04 were stained with Oil Red-O on day 8 of differentiation. The lipid accumulation was quantified as absorbance. (*n* = 5) Data are represented as mean ± SD. **(G)** Relative gene expression levels of 3T3-L1 cells treated with JIB-04 during differentiation (*n* = 4). Data are represented as mean ± SD. The black bars represent control and the white bars represent JIB-04. **(H)** H3K9me3 in the promoter region of Pparg was measured with ChIP-qPCR on day 4 during the differentiation of 3T3-L1 cells treated with 100 nM FK866 or 100 nM FK866 and 5 mM DM-αKG (*n* = 5). Data are represented as mean ± SD. **(I)** Relative gene expression levels of 3T3-L1 cells treated with 100 nM FK866 or 100 nM FK866, and 5 mM DM-αKG on day 4 of differentiation (*n* = 5). Data are represented as mean ± SD. **(J)** Oil Red-O staining of 3T3-L1 cells treated with 100 nM FK866 or with 100 nM FK866 and 5 mM DM-αKG on day 8 of differentiation. The lipid accumulation was quantified as absorbance. (*n* = 4). Ctrl represents control. *p < 0.05, **p < 0.01, ***p < 0.005.

It is well established that there is a sequential gene activation cascade activated by transcription factors as a key event controlling preadipocyte differentiation. In particular, C/EBPβ and C/EBPδ initiate the differentiation process and induce the expression of the master regulators of adipogenesis, PPARγ and C/EBPα, which go on to activate their adipogenic target genes (Farmer, 2006). We examined whether the expression of these transcription factors and their target genes during adipogenesis were influenced by NAD+ levels. While the initial adipogenic transcription factors, *Cebpb* and *Cebpd*, reached their peak expression levels regardless of FK866 treatment, the master regulators of adipogenesis, *Pparg* and *Cebpa*, and their target adipogenic genes, *Ap2*, *Adipoq*, and *Glut4*, were significantly suppressed by the reduced NAD+ levels (Figures 3A–C). In the cascade of gene regulation controlling adipogenesis, PPARγ and C/EBPα are thought to be directly induced by C/EBPβ and C/EBPδ. However, our results indicated that NAD+ must be upregulated to induce PPARγ and C/EBPα after the rise of C/EBPβ and C/EBPδ. These results suggest that NAD+ metabolism is involved in the induction of PPARγ and C/EBPα during adipogenesis in 3T3-L1 cells.

### α-Ketoglutarate-Dependent Demethylation of H3K9me3 at the *Pparg* Promoter Contributes to 3T3-L1 Preadipocyte Differentiation

We next investigated how NAD+ metabolism affected the expression of PPARγ and C/EBPα during adipogenesis. It has been demonstrated that PPARγ and C/EBPα expression is regulated through epigenetic mechanisms. Notably, demethylation of histone H3K9 in the promoter region of these genes has been shown to be critical for their efficient transcription. Generally, methylation status is determined by the balance between methylation and demethylation, which is maintained by histone methyltransferases and demethylases, respectively. It has been known that αKG, an intermediate metabolite in the TCA cycle, serves as a cofactor for histone demethylation through histone lysine demethylases (KDMs), and αKG levels regulate the methylation status of histones through KDMs (Carey et al., 2015). Interestingly, we revealed that the levels of αKG significantly increased during preadipocyte differentiation, corelating with NAD+ levels (Figures 2A, 3D), while FK866 treatment abolished the rise of αKG levels after differentiation was induced (Figures 2D, 3E). Therefore, we hypothesized that enhanced NAD+ synthesis regulates the transcription of *Pparg* and *Cebpa* through αKG-dependent demethylation and promotes the differentiation of 3T3-L1 preadipocytes. First, we examined whether the KDM inhibition suppressed 3T3-L1 preadipocyte differentiation by treating cells with JIB-04, a pan-inhibitor of jumonji demethylases (Wang et al., 2013). As expected, JIB-04 treatment suppressed the differentiation of 3T3-L1 cells (Figure 3F). Additionally, the expression of *Pparg* and *Cebpa*, but not *Cebpb*, were significantly inhibited by JIB-04 treatment (Figure 3G). Next, we estimated the methylation status of histone H3K9 at the *Pparg* promoter region using ChIP-qPCR. Consistent with the previous report, H3K9me3 levels were significantly reduced during differentiation, while FK866 treatment blocked this reduction (Figure 3H). Next, we investigated whether exogenous supplementation of dimethyl alpha-ketoglutarate (DM-αKG), a cell membrane permeable derivative of αKG, could rescue these defects caused by FK866 treatment. We confirmed that DM-αKG supplementation fully restored αKG levels in the presence of FK866 (Figure 3E). Importantly, DM-αKG supplementation rescued the demethylation of H3K9me3, *Pparg* expression, and the differentiation of 3T3-L1 cells, all of which were suppressed by FK866 treatment (Figures 3H–J). Together, these data demonstrate that αKG levels, boosted by NAD+, are necessary for the demethylation of H3K9me3 at the *Pparg* promoter region and lead to 3T3-L1 preadipocyte differentiation.

### Sirtuins and PARPs Regulate Preadipocyte Differentiation Independently of NAD+ Levels

In addition to serving as a cofactor, NAD+ also functions as a substrate for sirtuins and PARPs, which have been reported to be involved in adipogenesis. Therefore, we investigated whether NAD+ affected preadipocyte differentiation through sirtuin- and/or PARP-mediated mechanisms. Among seven sirtuin homologs in mammals, SIRT1, SIRT6, and SIRT7 are localized to the nucleus. Thus, we silenced these nuclear sirtuins in 3T3-L1 cells using small interfering RNA (siRNA) and investigated the effects on differentiation. Knockdown of SIRT1 or SIRT6 in 3T3-L1 preadipocytes significantly suppressed differentiation, whereas knockdown of SIRT7 enhanced differentiation (Supplementary Figures 1A–C), indicating that different sirtuins can have different effects on differentiation. Subsequently, we assessed the global acetylation level of histone H3K9, an important residue controlling chromatin state, of 3T3-L1 cells and found it to be unchanged when NAD synthesis was blocked by FK866 treatment (Supplementary Figure 1D). These results suggest that sirtuins are important for adipogenesis, but are not regulated by NAD+ levels. In contrast, FK866 treatment significantly reduced poly(ADP-ribosyl)ation during preadipocyte differentiation (Supplementary Figure 2A). However, the pharmacological inhibition of PARP1, PARP2, or both PARP1 and PARP2 by AG-14361, UPF-1069, or Olaparib, respectively, had little effect on lipid accumulation; however, there was an exception when used at a very high concentration (Supplementary Figures 2B–D). Although olaparib significantly suppressed poly(ADP-ribosyl)ation during differentiation of 3T3-L1 preadipocytes adipogenic gene expression was not changed, even under a very high concentration of Olaparib (Supplementary Figures 2E,F). Together, our data suggest that PARPs and sirtuins may have roles in adipogenesis, but are not regulated by NAD+ levels.

### DM-αKG Supplementation Prevents Diet-Induced Obesity in Mice by Promoting Appropriate Adipogenesis

**FIGURE 4:**
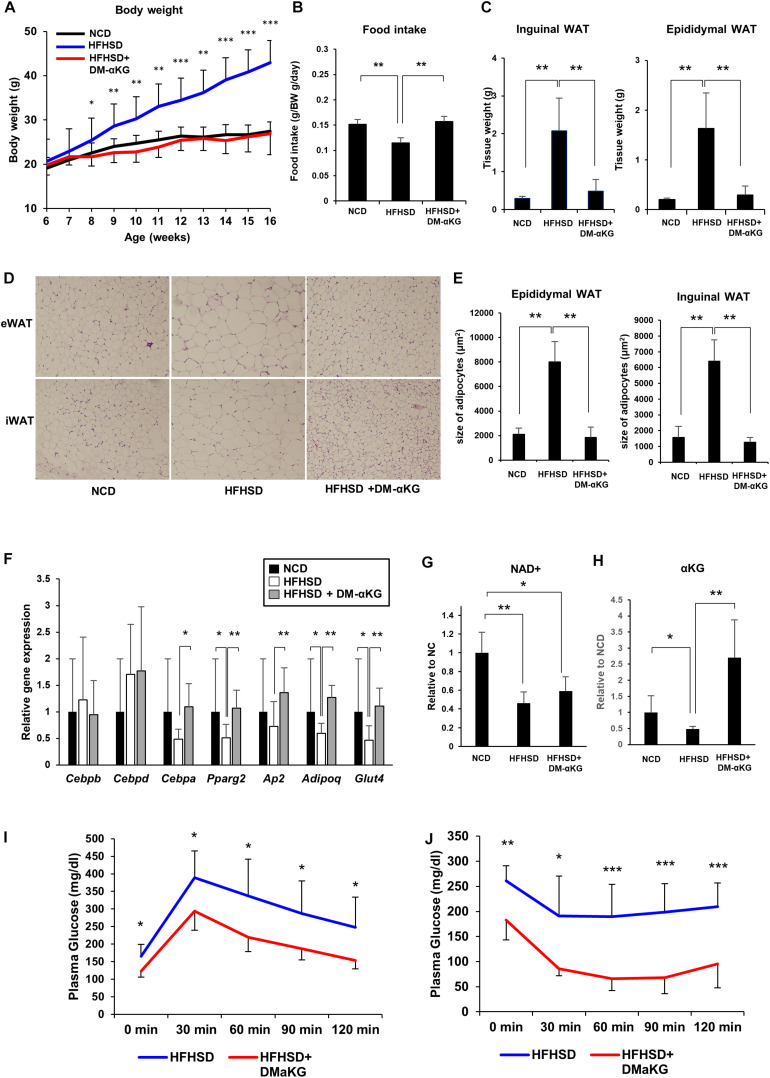
DM-αKG supplementation prevents diet-induced obesity in mice by promoting appropriate adipogenesis. **(A)** Body weight of C57BL/6J mice fed a normal chow diet (NCD) or high fat high sucrose diet (HFHSD) (*n* = 4–6). 1.5% dimethyl α-ketoglutarate (DM-αKG) was supplemented in the drinking water. Data are represented as mean ± SD. **(B)** The average of food intake of each group was measured weekly for the first 4 weeks and was adjusted by body weight. Data are represented as mean ± SD. (*n* = 4). **(C)** The weight of white adipose tissue was measured at 16 weeks of age. Data are represented as mean ± SD. (*n* = 4–6). **(D)** Representative images of paraffin sections of white adipose tissue at 16 weeks old stained with hematoxylin and eosin (H&E). Images were acquired with an Olympus BX61, 10×. (*n* = 2). **(E)** Adipocyte size in H&E stained samples was calculated using ImageJ. (*n* = 8) Data are represented as mean ± SD. **(F)** Relative expression levels of genes related to adipogenesis in epididymal white adipose tissue (eWAT) at 16 weeks old (*n* = 6). Data are represented as mean ± SD. **(G)** The level of NAD+ in eWAT of wild type mice fed NCD, HFHSD, or HFHSD with DM-αKG were measured after 10 weeks of HFHSD with LC-MS. Data are represented as mean ± SD. (*n* = 4). **(H)** The level of α-KG in blood plasma of wild type mice fed NCD, HFHSD, or HFHSD with DM-αKG were measured after 10 weeks of HFHSD with GC-MS. Data are represented as mean ± SD. (*n* = 4). **(I)**, **(J)** The plasma glucose level in intraperitoneal glucose tolerance test after 10 weeks of HFHSD **(I)** and in intraperitoneal insulin tolerance test after 16 weeks of HFHSD **(J)**. Data are represented as mean ± SD (*n* = 5–6). *p < 0.05, **p < 0.01, ***p < 0.005.

Finally, we attempted to elucidate the physiological significance of the NAD+-αKG axis during obesity. Mice fed a high-fat high-sucrose diet (HFHSD) were orally administered DM-αKG in drinking water. Surprisingly, DM-αKG administration completely suppressed HFHSD-induced obesity, resembling a normal diet (Figure 4A). We also found that food intake was increased in the DM-αKG administered group (Figure 4B). The weight of adipose tissue was also drastically reduced in the DM-αKG administered group (Figure 4C). We also examined the morphology of adipose tissues. HFHSD caused hypertrophy of adipocytes, while the size of adipocytes in the DM-αKG administered group was significantly smaller than those of the HFHSD fed group (Figures 4D,E). Additionally, we found adipogenic gene expression to be impaired in the HFHSD fed mice, which were rescued by DM-αKG administration (Figure 4F). Then we assessed the level of NAD+ and αKG to determine whether the level of these metabolites affected the morphology of adipocytes and the expression of genes involving adipogenesis. The levels of NAD+ in the epididymal white adipose tissue (eWAT) was significantly reduced by HFHSD feeding (Figure 4G). Accordingly, the level of αKG in the blood plasma was also reduced in HFHSD fed mice, indicating systemic metabolic state was altered by HFHSD feeding (Figure 4H). The administration of DM-αKG increased the level of αKG in the blood plasma without altering NAD+ levels in eWAT (Figures 4G,H). These results suggest that the metabolic change caused by HFHSD deteriorates the proper preadipocyte differentiation and leads to the hypertrophy of adipocytes and that the supplementation of αKG is important for reconstructing the proper preadipocyte differentiation. Importantly, the glucose intolerance and insulin resistance caused by HFHSD feeding were significantly improved in DM-αKG administered group (Figures 4I,J), suggesting the supplementation of αKG would possibly become a therapeutic option for obesity and subsequent glucose intolerance.

## Discussion

**FIGURE 5:**
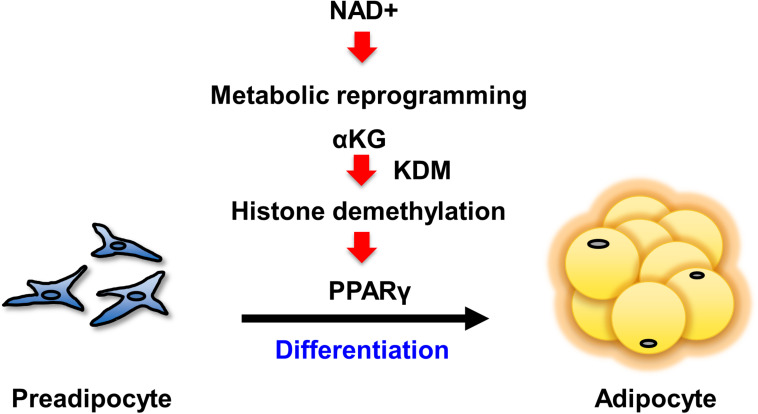
Scheme of the mechanism by which NAD+ metabolism regulates preadipocyte differentiation through epigenetics.

Our present study has demonstrated that enhancing NAD+ synthesis in the salvage pathway is a critical event for the differentiation of 3T3-L1 preadipocytes. The increased levels of NAD+ promoted both aerobic and anaerobic glycolysis, followed by the TCA cycle and mitochondrial respiration. Specifically, the increased αKG contributed to the demethylation of histone H3K9me3 in the promoter region of *Pparg*. This demethylation epigenetically activated *Pparg* transcription, leading to cellular differentiation (Figure 5). Although it had been generally assumed that the metabolic changes that occur during differentiation were simply an adaptive result of cell specialization (Cummins et al., 2014), a number of recent studies have demonstrated that metabolic changes actually determine the fate of undifferentiated progenitor or stem cells (Folmes et al., 2011). In embryonic stem cells (ESCs) or induced pluripotent stem cells (iPSCs), the metabolic shift from anaerobic to aerobic glycolysis is an important cue for differentiation (Xu et al., 2013). In hematopoietic stem cells, metabolic reprogramming from cytosolic glycolysis to mitochondrial metabolism, such as oxidative phosphorylation and the TCA cycle, is a key event controlling the induction of differentiation (Takubo et al., 2013; Mathieu et al., 2014; Prigione et al., 2014). Additionally, increased activity of the TCA cycle and glycolysis has been reported in obesity (Cummins et al., 2014). Thus, our results also support the idea that metabolic changes critically regulate cellular differentiation processes.

We identified the αKG-mediated demethylation of histone H3K9me3 in the *Pparg* promoter to be the downstream event of increased NAD+ levels during 3T3-L1 preadipocyte differentiation. Previously, SETDB1, a histone methyltransferase (KMT), was shown to be involved in preadipocyte differentiation (Matsumura et al., 2015). The repressive histone mark, H3K9me3, in the promoter region of *Pparg* and *Cebpa* prevented C/EBPβ from binding to this region. However, the downregulation of SETDB1 in the early phase of differentiation diminished the methylation of H3K9me3, leading to active transcription of *Pparg* and *Cebpa* (Matsumura et al., 2015). Given that histone methylation status is regulated by the interplay between KMTs and KDMs, it is possible that KDMs cooperate with KMTs to determine the methylation status of the *Pparg* promoter. αKG is known to be a cofactor for histone demethylation by KDMs and for DNA demethylation by the ten eleven translocation (TET) enzyme, and αKG levels have been reported to affect methylation levels (Carey et al., 2015; Yang et al., 2016). Thus, the increased level of αKG is a reasonable explanation for how metabolic changes contribute to preadipocyte differentiation. However, additional studies are needed to determine the specific type of KDM responsible for demethylating histone H3K9me3 in the *Pparg* promoter.

We demonstrate that only the high concentration of PARP inhibitors suppressed the differentiation of 3T3-L1 preadipocytes, but the effect to gene expression was relatively limited compared to the inhibition of NAD+ synthesis (Supplementary Figure 2). Previous works have demonstrated poly(ADP-Ribosyl)ation (PARylation) by PARP to be involved in adipocyte differentiation. However, the contribution of PARP to adipogenesis appears to be complex (Szántó and Bai, 2020). One group has shown the PARylation of C/EBPβ to suppress its binding to target genes, which suppresses differentiation of 3T3-L1 preadipocytes (Luo et al., 2017). Meanwhile, other groups have shown PARP1 or PARP2 to promote adipocyte differentiation by cooperating with PPARγ(Bai et al., 2007; Fujiki et al., 2013). Ryu et al. (2018) demonstrated that NAD+ levels differentially regulated between the nucleus and the cytoplasm coordinately controls adipocyte differentiation. In the early phase of differentiation, decreased NAD+ levels in the nucleus promoted C/EBPβ transcription by suppressing PARP1 activity. Subsequently, increased NAD+ levels in the cytosol supported the late phase of adipogenesis by enhancing glucose metabolism (Ryu et al., 2018). A very recent study has also indicated that Nmnat1 inhibits adipogenesis by promoting histone H2B Glu35 ADP-rybosilation through activation of PARP1 (Huang et al., 2020). Thus, PARP suppresses differentiation in the early phase though C/EBPβ, but facilitates differentiation in the later stage by cooperating with PPARγ. Additionally, the enhancement of glucose metabolism is important event in the late phase of 3T3-L1 preadipocytes differentiation. Nevertheless, our results indicate that NAD+ upregulation profoundly contributes to preadipocyte differentiation, independently of PARylation. We found NAD+ levels were extremely low in the early phase compared to the later phase. These marginal changes in NAD+ levels may not affect PARP activity. However, involvement of NAD+ metabolism in adipogenesis may vary according to timing and cellular compartment and further studies are necessary to reveal complex role of NAD+-PARP interaction during adipogenesis.

A number of studies have shown sirtuins to be associated with adipocyte differentiation. Originally, SIRT1 has been reported to suppress adipocyte differentiation and subsequent studies also demonstrated the inhibitory effects of SIRT1 in adipogenesis (Picard et al., 2004; Jing et al., 2007; Mayoral et al., 2015; Zhou et al., 2016; Chen et al., 2017; Fang et al., 2017). However, the oppose effects of SIRT1 in the adipogenesis was also reported (Qiao and Shao, 2006). Other nuclear surtuins, SIRT6 and SIRT7, reportedly promote adipocyte differentiation (Picard et al., 2004; Jing et al., 2007; Chen et al., 2017; Fang et al., 2017). SIRT6 is essential for mitotic clonal expansion of 3T3-L1 preadipocyte (Picard et al., 2004; Jing et al., 2007; Chen et al., 2017; Fang et al., 2017). SIRT7 promotes adipogenesis by inhibiting autocatalytic activation of SIRT1 (Picard et al., 2004; Jing et al., 2007; Chen et al., 2017; Fang et al., 2017). In addition, SIRT2 also localized in nucleus and suppress adipocyte differentiation (Picard et al., 2004; Jing et al., 2007; Wang and Tong, 2009; Chen et al., 2017; Fang et al., 2017). Thus, each sirtuin has opposing functions during adipocyte differentiation. Indeed, our results also indicated silencing of each sirtuin homolog had opposing effects in adipogenesis. Therefore, it seems unreasonable for the activities of sirtuins to be regulated by NAD+ levels during adipocyte differentiation because increased NAD+ levels may activate all sirtuins. We assume that activities of sirtuins is not regulated by NAD+ levels, but rather by the level of sirtuins itself during preadipocyte differentiation.

Many studies reported that enhancing NAD+ level by the administration of NAD+ precursors, including NMN and NR, ameliorated obesity and its related metabolic dysfunctions. In this study, we demonstrated that administration of αKG also suppresses the obesity and glucose intolerance induced by HFHSD. In this respect, it is possible that the beneficial effect of supplementation of NAD+ precursors is partially attributed to the metabolic compensation for the differentiation of adipocytes. Recently, several studies demonstrated the beneficial effects of αKG supplementation. It has been reported that αKG administration extends the adult lifespan of *C. elegans* by ATP synthase-TOR axis (Chin et al., 2014). Long-term supplementation of αKG also extends the healthspan in mice by reducing the chromic inflammation (Asadi Shahmirzadi et al., 2020). αKG supplementation also improved the energy expenditure by promoting UCP1 expression (Tian et al., 2020). NAD+ abundantly exists in mitochondria and support TCA cycle and oxidative phosphorylation. αKG administration may stimulates mitochondrial energy metabolism independently of demethylation-mediated gene expression. The beneficial effects αKG may be complex and are not limit to adipose tissue. Further investigations are necessary to elucidate the effects of αKG on the other organs and the systemic metabolism.

In conclusion, this study revealed a novel insight regarding the mechanisms of preadipocyte differentiation which involves NAD+-dependent metabolic reprogramming and subsequent epigenetic alterations. Although further studies are required to explore the effect of metabolic reprogramming on the global epigenetic landscape and the role of this mechanism in adipogenesis *in vivo*, our results indicate that the NAD+-αKG axis is a potential therapeutic target for obesity-related metabolic disorders.

## Data Availability Statement

The raw data supporting the conclusions of this article will be made available by the authors, without undue reservation.

## Ethics Statement

The animal study was reviewed and approved by the Animal Experiment Committee of the University of Toyama.

## Author Contributions

TN, KO, and KT conceived and designed the experiments. KO, AN, YN, and TN performed the experiments and analyzed the data. AN, YN, KY, and IU contributed to reagents, materials, and analysis tools. KO and TN wrote the manuscript. All authors reviewed the manuscript.

## Conflict of Interest

The authors declare that the research was conducted in the absence of any commercial or financial relationships that could be construed as a potential conflict of interest.
