# Supplementary material for: NAD+ Metabolism Regulates Preadipocyte Differentiation by Enhancing α-Ketoglutarate-Mediated Histone H3K9 Demethylation at the PPARγ Promoter
Source: Front Cell Dev Biol. 2020 Nov 24;8:586179. doi: 10.3389/fcell.2020.586179 (PMC7732485; doi:10.3389/fcell.2020.586179)
Supplement: Supplementary file 1 [file Data_Sheet_1.PDF]

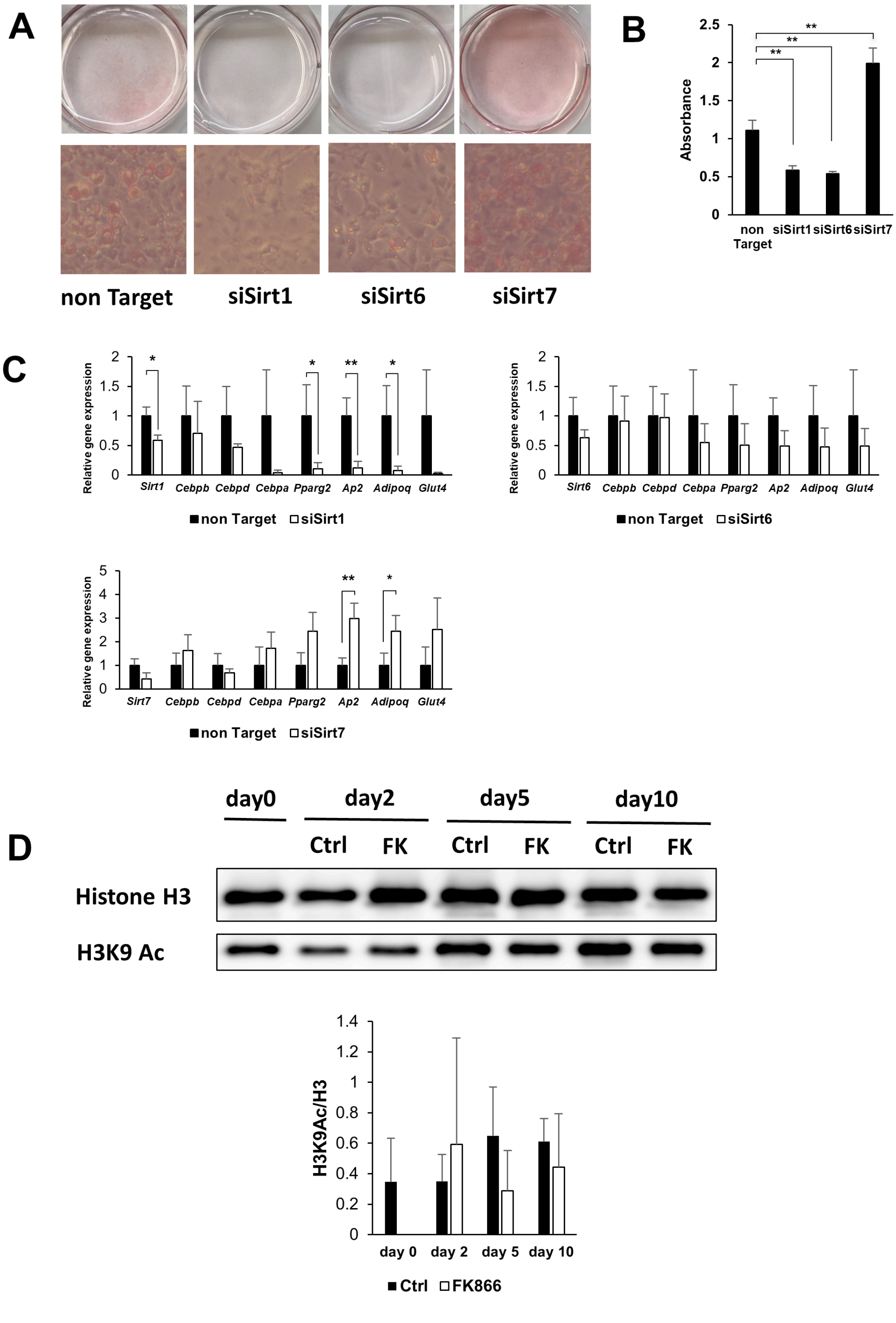

Figure S1. Sirtuins regulate preadipocyte differentiation independently of NAD+.

(A and B) 3T3-L1 preadipocytes were treated with siRNA and Oil Red-O stained on day 8 of differentiation. (A) The stained lipid droplets were quantified (n=3). (B) Data are represented as mean ± SD. **(C)** Relative gene expression levels of 3T3-L1 cells on day 5 of differentiation (n=3). Data are represented as mean ± SD. **(D)** Western Blotting of 3T3-L1 cells treated with FK866 during differentiation. The representative result from 3 independent experiments is shown. The signals of H3K9 Ac were quantified and adjusted with those of Histone H3 (n=3). Ctrl represents control.

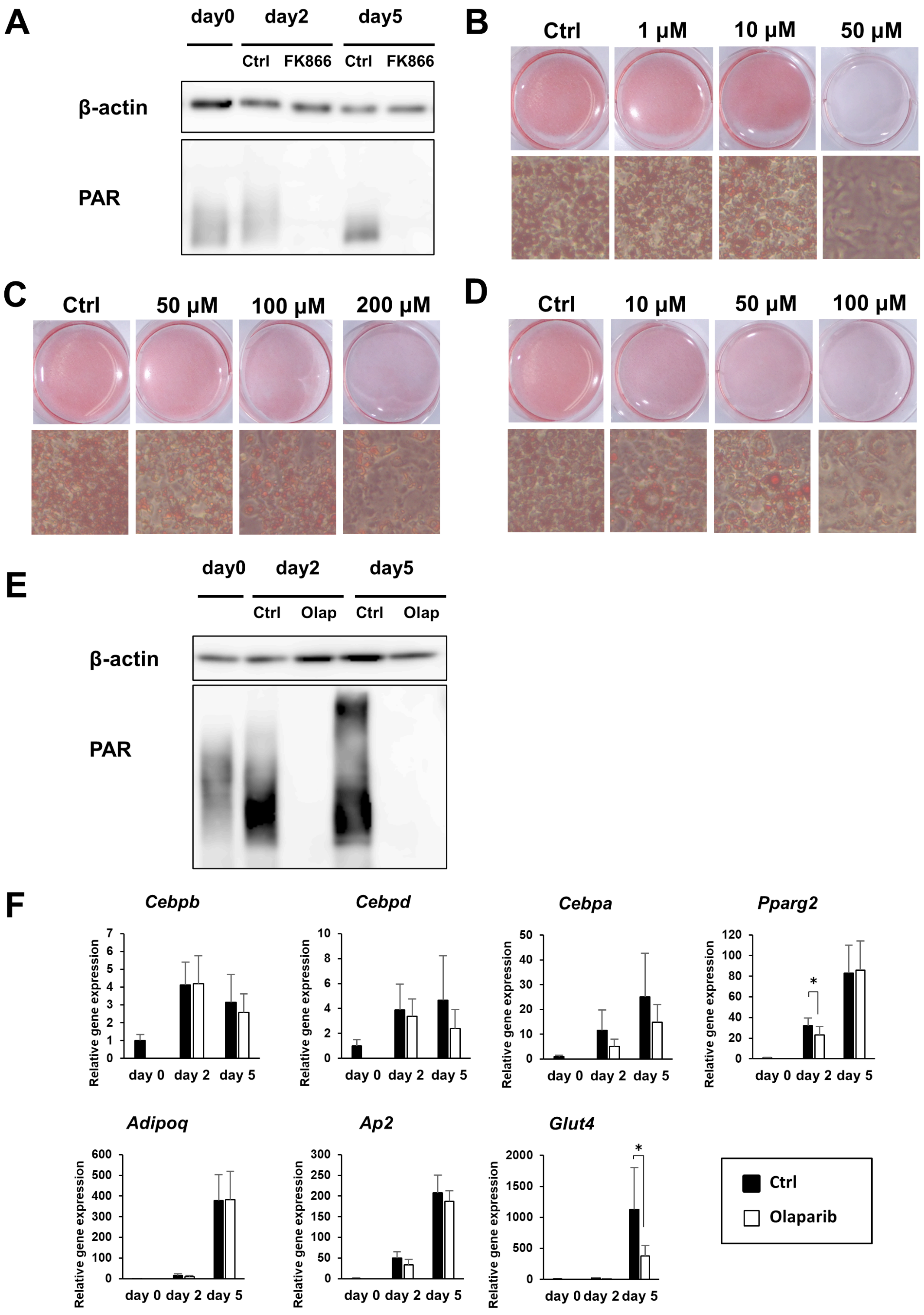

Figure S2. PARPs regulate preadipocyte differentiation independently of NAD+
(A) Western Blotting of PAR in 3T3-L1 cells treated with FK866 during differentiation. The representative result from 3 independent experiments is shown. PAR represents poly ADP-ribose. Ctrl represents control. **(B-D)** Oil Red-O staining of 3T3-L1 cells on day 8 of differentiation. The cells were treated with AG-14361 (B), UPF-1069 (C), or Olaparib (D) at the indicated concentration from day 0 to day 5. **(E)** Western Blotting of PAR in 3T3-L1 cells treated with 100 μM Olaparib. **(F)** Relative gene expression levels of 3T3-L1 cells treated with 100 μM Olaparib. (n=4). Data are represented as mean ± SD.

Table S1. Primers used in this study.

Target	Sequence
Rpl13a_F	AGCGCCTCAAGGTGTTGGA
Rpl13a_R	GAGTGGCTGTCACTGCCTGGTA
Nampt_F	CCTGTTCCAGGCTATTCTGTTC
Nampt_R	TCATGGTCTTTCCCCCAAGC
Nmnat1_F	GTGGAGACTGTGAAGGTGCTC
Nmnat1_R	GTGAGCTTTGTGGGTAACTGC
Hk2_F	GGAACCCAGCTGTTTGACCA
Hk2_R	CAGGGGAACGAGAAGGTGAAA
Pkm_F	GCCGCCTGGACATTGACTC
Pkm_R	CCATGAGAGAAATTCAGCCGAG
Pkha1_F	GAAATGTGACCTTCATCGGCT
Pkha1_R	TGATCCGCCTTTAGCTCCATC
Sdha_F	CTTGAATGAGGCTGACTGTG
Sdha_R	ATCACATAAGCTGGTCCTGT
Cs_F	GGACAATTTTCCAACCAATCTGC
Cs_R	TCGGTTCATTCCCTCTGCATA
Mdh2_F	TTGGGCAACCCCTTTCACTC
Mdh2_R	GCCTTTCACATTTGCTCTGGTC
Idh3a_F	TGGGTGTCCAAGGTCTCTC
Idh3a_R	CTCCCACTGAATAGGTGCTTTG
Ogdh_F	AGGGCATATCAGATACGAGGG
Ogdh_R	CTGTGGATGAGATAATGTCAGCG
Cebpb_F	CTGCGGGGTTGTTGATGT
Cebpb_R	ATGCTCGAAACGGAAAAGGT
Cebpd_F	CGACTTCAGCGCCTACATTGA
Cebpd_R	CTAGCGACAGACCCCACAC
Cebpa_F	CAAGAACAGCAACGAGTACCG
Cebpa_R	GTCACTGGTCAACTCCAGCAC
Pparg2_F	TCGCTGATGCACTGCCTATG
Pparg2_R	GAGAGGTCCACAGAGCTGATT
Ap2_F	AAGGTGAAGAGCATCATAACCCT
Ap2_R	TCACGCCTTTCATAACACATTCC
Adipoq_F	TGTTCCTCTTAATCCTGCCCA
Adipoq_R	CCAACCTGCACAAGTTCCCTT
Glut4_F	GTGACTGGAACACTGGTCCTA
Glut4_R	CCAGCCACGTTGCATTGTAG
Sirt1_F	TGATTGGCACCGATCCTCG
Sirt1_R	CCACAGCGTCATATCATCCAG
Sirt6_F	ATGTCGGTGAATTATGCAGCA
Sirt6_R	GCTGGAGGACTGCCACATTA
Sirt7_F	GGGTCCTAATGGAGTATGGACA
Sirt7_R	CTCATGCAAACGGGTGATGC
Pparg promoter_F	GGCCAAATACGTTTATCTGGTG
Pparg promoter_R	GTGAGGGGCGTGAACTGTA
